# Modeling lamellar disruption within the aortic wall using a particle-based approach

**DOI:** 10.1038/s41598-019-51558-2

**Published:** 2019-10-25

**Authors:** H. Ahmadzadeh, M. K. Rausch, J. D. Humphrey

**Affiliations:** 1 Department of Biomedical Engineering, Yale University, New Haven, CT USA; 2 Department of Aerospace Engineering and Engineering Mechanics, Department of Biomedical Engineering, University of Texas at Austin, Austin, TX USA

**Keywords:** Computational biophysics, Biomedical engineering

## Abstract

Aortic dissections associate with medial degeneration, thus suggesting a need to understand better the biophysical interactions between the cells and matrix that constitute the middle layer of the aortic wall. Here, we use a recently extended “Smoothed Particle Hydrodynamics” formulation to examine potential mechanisms of aortic delamination arising from smooth muscle cell (SMC) dysfunction or apoptosis, degradation of or damage to elastic fibers, and pooling of glycosaminoglycans (GAGs), with associated losses of medial collagen in the region of the GAGs. First, we develop a baseline multi-layered model for the healthy aorta that delineates medial elastic lamellae and intra-lamellar constituents. Next, we examine stress fields resulting from the disruption of individual elastic lamellae, lost SMC contractility, and GAG production within an intra-lamellar space, focusing on the radial transferal of loading rather than on stresses at the tip of the delaminated tissue. Results suggest that local disruptions of elastic lamellae transfer excessive loads to nearby intra-lamellar constituents, which increases cellular vulnerability to dysfunction or death. Similarly, lost SMC function and accumulations of GAGs increase mechanical stress on nearby elastic lamellae, thereby increasing the chance of disruption. Overall these results suggest a positive feedback loop between lamellar disruption and cellular dropout with GAG production and lost medial collagen that is more pronounced at higher distending pressures. Independent of the initiating event, this feedback loop can catastrophically propagate intramural delamination.

## Introduction

Aortic dissection is a life-threatening condition characterized by blood entering the medial layer of the aortic wall via a so-called false lumen, which may or may not thrombose. Such a false lumen can form due to either an intimal defect or an intramural delamination that propagates and connects with the lumen. Despite considerable data from histopathology^1–3^, medical imaging^4,5^, and mechanical testing^6–8^, the biomolecular and biophysical mechanisms by which aortic dissection develops remain poorly understood^9–11^. Nevertheless, aortas susceptible to dissection tend to exhibit medial degeneration, as evidenced by fragmentation or loss of elastic fibers, dysfunction or apoptosis of smooth muscle cells (SMCs), localized pooling of glycosaminoglycans (GAGs), and disorganization or remodeling of collagen^12,13^. Of course, regardless of the specific histopathology in individual cases, dissection occurs when wall stress exceeds wall strength locally, hence there is a pressing need for detailed micro-scale biomechanical analyses.

There is now a considerable literature on the mechanics of aortic dissection^14^. Using peeling tests on samples of human aorta, Holzapfel and colleagues^7,15^ identified relationships between in-plane anisotropic properties of the aortic wall and the direction of propagation of a dissection. In further studies, Gültekin *et al*.^16–18^ simulated tear propagation within aortic tissues by using phase-field modeling. Their experimentally-calibrated and -validated simulations investigated the effects of various factors such as failure criteria and tissue anisotropy on the direction of the dissection propagation within aortic wall. Using biaxial testing, Sugita and Matsumoto^19,20^ found a relation between the direction of the damage propagation and local orientations of the collagen fibers. In contrast, Vorp and colleagues^6,21,22^ focused on the role of radially-oriented matrix fibers that connect medial layers and thus serve to resist intra-lamellar separation. Barocas and colleagues^23,24^ similarly reported biomechanical data and model predictions that focus on fibrous components within the wall, including intra-lamellar connections. Hence, most prior studies have either used traditional continuum mechanical approaches or focused primarily on the role of matrix fibers that endow the wall with significant stiffness and strength. Notwithstanding the importance of such studies, there has been little attention to the three most common histopathological features associated with aortic dissection^1–3^, namely, disruption of individual elastic layers, local dysfunction or apoptosis of SMCs, and pooling of GAGs, with associated losses of medial collagen.

Because of our different focus, we necessarily employ a different approach to model the local biophysical mechanisms of aortic dissection – our recently developed data-driven, extended smoothed particle hydrodynamics (SPH) framework that allows one to study local effects while interpreting results in terms of continuum quantities such as stress^25,26^. We thereby can study potentially separate or collective roles of localized defects in elastin integrity, loss of SMC contractility or outright cell drop-out, and the pooling of GAGs. Given the increasing use of mouse models of thoracic aortopathy^27,28^, we use murine rather than human data to inform our model. Note, therefore, that the medial layer of the normal thoracic aorta in the mouse consists of elastic fibers organized into ~ six nearly concentric elastic laminae that delimit ~ five separate layers of SMCs that are embedded within fibrillar collagens and diffuse GAGs^29–31^. Normal mass fractions of the medial constituents in mice are roughly 32% for elastin, collagen, and smooth muscle, with GAGs normally occupying ~4% or less^32^. In cases of medial degeneration, however, GAG content can increase significantly, particularly within localized pools, and smooth muscle function or presence can decrease significantly^33,34^. Most computational models of the normal arterial wall have assumed homogenized properties, even if within the context of a structurally-motivated constitutive relation or a constrained mixture model of constituent turnover^35,36^. In contrast, we present a model of the aorta that naturally separates the wall into its multi-layered structure, including the five separate lamellar units of the media of appropriate dimensions, and accounts for localized defects on the scale of a few microns that are motivated by specific histopathology from mouse models of aortic dissection^34,37,38^.

## Methods

### SPH framework

Let an idealized circular cross-section of the descending thoracic aorta of a mouse be defined in a passive homeostatic reference configuration by inner and outer radii *R*_in_ = 646.8 μm and *R*_out_ = 687.0 μm (with wall thickness *H* = 40.2 μm), for which luminal pressure is 102 mmHg and axial stretch is 1.62^39^. In response to changing boundary conditions, particles at any position within this reference configuration (*R*, *θ*, *Z*) can be mapped to positions within any non-homeostatic current/deformed configuration given by radial, circumferential, and axial coordinates (*r*, *θ*, *z*). To focus on local defects, we consider a domain defined by a 90° sector with periodic boundary conditions, which also reduces computational costs. Mirroring the microstructural composition of the aorta that is revealed by histology^39^, the wall is divided into a media that occupies two-thirds (an inner layer with thickness *H*_M_ = 28.4 μm) and an adventitia that occupies one-third (an outer layer with thickness *H*_A_ = 11.8 μm) of the wall in the homeostatic configuration; we do not include an intimal layer, which is thought to be mechanobiologically critical under flow but mechanically negligible in a healthy murine aorta. The media is further divided into six repeating layers of elastic fibers (or lamellae) that are lined with fibrillar collagen and that separate single layers of SMCs and diffuse aggregating GAGs; these SMCs can contract and relax as appropriate, noting that loss of smooth muscle contractility is also a distinguishing feature of thoracic aortopathies^9–11^. The adventitia, in contrast, consists primarily of a dense network of fibrillar collagen with modest elastin but no SMCs or aggregating GAGs.

**Figure 1:**
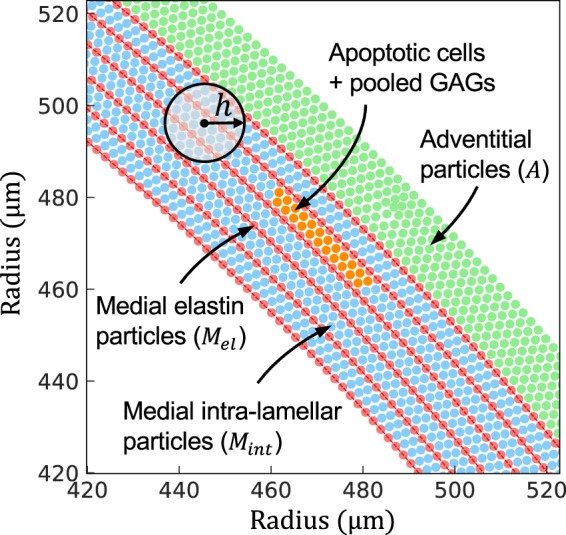
Particle arrangement in the SPH aortic model. Particles are categorized into three groups: medial elastin (*M*_*el*_-red), medial intra-lamellar (*M*_*int*_-blue), and adventitial (*A*-green). Also shown is a collection of intra-lamellar particles (orange) undergoing apoptosis and loss of matrix and replacement with GAGs. The kernel support denoted by *h* determines the number of particles included in each list of neighbors, which allows local smoothing and calculation of continuum quantities from individual particle forces and motions. Particle density is 0.24 per *μm*^2^, with individual particle spacing ~2 μm.

We model the mechanics of this aortic wall using our extended SPH formulation for nonlinear biosolids, details of which can be found elsewhere^25,26^, including verification and validation studies using analytical and standard finite element solutions, including damage mechanics. Briefly, we represent the computational domain of the aortic wall with an arrangement of individual particles that carry information on their surrounding volume and yield, via appropriate local smoothing, mechanical quantities such as stress. We use ***X***_*i*_ and ***x***_*i*_ to refer to referential and current positions of the particle labelled by *i*. Each particle has a set (denoted by *S*) of neighboring particles from which the numerical approximation of any physical field quantity (denoted generically by *g*) is obtained as

1 $$\hat{g}({{\boldsymbol{X}}}_{i})=\sum _{j\,\in S}\,g({{\boldsymbol{X}}}_{j}){V}_{j}\xi ({R}_{j}),$$

where $\hat{g}({X}_{i})$ is the approximated value of a scalar field *g*(***X***) at particle *i* based on values at neighboring locations *j*, where *V*_*j*_ is the volume associated with particle *j*, and *ξ*(*R*_*j*_) is a kernel function that governs interactions amongst particles depending on the length of their reference distance vector (*R*_*j*_ = |***R***_*j*_| = |***X***_*j*_−***X***_*i*_|). Following our previous work^25,26^, we use a Spiky kernel, a third-order polynomial function that can be written

2 $$\xi ({R}_{j})=\{\begin{array}{cc}\frac{10{(h-{R}_{j})}^{3}}{\pi {h}^{5}} & {R}_{j} < h\\ 0 & {R}_{j}\ge h\end{array},$$

where *h* is the kernel support (Fig. 1).

Referential gradients of the approximated scalar field are calculated by

3 $${\nabla }_{{\boldsymbol{X}}}\hat{g}({{\boldsymbol{X}}}_{i})=\sum _{j\,\in S}\,g({{\boldsymbol{X}}}_{j}){V}_{j}\,{\nabla }_{{\boldsymbol{X}}}\xi ({R}_{j}),$$

where the gradient of the kernel function is

4 $${\nabla }_{{\boldsymbol{X}}}\xi ({R}_{j})=(\frac{\partial \xi ({R}_{j})}{\partial {R}_{j}})\frac{{{\boldsymbol{R}}}_{j}}{{R}_{j}}.$$

Following^26,40^, zeroth-order and first-order completeness of numerical approximations of these derivatives are satisfied as following. First, we symmetrize the gradient of the kernel function

5 $${\nabla }_{{\boldsymbol{X}}}\hat{g}({{\boldsymbol{X}}}_{i})=\sum _{j\,\in S}(g({{\boldsymbol{X}}}_{j})-g({{\boldsymbol{X}}}_{i})){V}_{j}\,{\nabla }_{{\boldsymbol{X}}}\xi ({R}_{j});$$

second, we introduce a correction matrix (denoted by ***A***_*i*_) in the gradient of the kernel function to obtain a corrected gradient ${\tilde{\nabla }}_{{\boldsymbol{X}}}\xi ({R}_{j})$ as

6 $${\tilde{\nabla }}_{{\boldsymbol{X}}}\xi ({R}_{j})={{\boldsymbol{A}}}_{i}^{-1}{\nabla }_{{\boldsymbol{X}}}\xi ({R}_{j})\,{\rm{with}}\,{{\boldsymbol{A}}}_{i}=\sum _{j\,\in S}\,{V}_{j}\,{\nabla }_{{\boldsymbol{X}}}\xi ({R}_{j})\otimes \,{{\boldsymbol{R}}}_{j}.$$

The deformation gradient at each particle *i*, that is ***F***_*i*_ = ∂***x***_*i*_/∂***X***_*i*_, is then computed as

7 $${{\boldsymbol{F}}}_{i}=\sum _{j\,\in S}\,{{\boldsymbol{r}}}_{j}\otimes {V}_{j}\,{\tilde{\nabla }}_{{\boldsymbol{X}}}\xi ({R}_{j}),$$

where ***r***_*j*_ = ***x***_*j*_−***x***_*i*_ is the distance between two particles in a current configuration.

Assuming hyperelastic material behavior, with a strain energy function *W*, the first Piola-Kirchhoff stress tensor (***P***_*i*_) and Cauchy stress tensor (***σ***_*i*_) at particle *i* are

8 $${{\boldsymbol{P}}}_{i}=2{{\boldsymbol{F}}}_{i}\frac{\partial W({{\boldsymbol{C}}}_{i})\,}{\partial {{\boldsymbol{C}}}_{i}},\,{{\boldsymbol{\sigma }}}_{i}=\frac{1}{{J}_{i}}{{\boldsymbol{P}}}_{i}{{\boldsymbol{F}}}_{i}^{T},$$

where ${{\boldsymbol{C}}}_{i}={{\boldsymbol{F}}}_{i}^{T}{{\boldsymbol{F}}}_{i}$ is the right Cauchy-Green tensor and ${J}_{i}=\det ({{\boldsymbol{F}}}_{i}^{2D}){\lambda }_{zi}$ is the volume change associated with particle *i*, here computed via area changes in the two-dimensional cross-section of the artery and the fixed axial stretch applied to the vessel (*λ*_*zi*_). Because the method is fundamentally particle based, we must compute forces needed to satisfy Newton’s second law of motion. The first Piola-Kirchhoff stress tensor (***P***_*i*_) is used to compute internal forces acting between particles as

9 $${{\boldsymbol{f}}}_{i}^{{\rm{int}}}=\sum _{j\,\in S}\,{{\boldsymbol{f}}}_{ij}=\sum _{j\,\in S}\,{V}_{i}{V}_{j}({{\boldsymbol{P}}}_{i}{\tilde{\nabla }}_{{\boldsymbol{X}}}\xi ({R}_{j})-{{\boldsymbol{P}}}_{j}{\tilde{\nabla }}_{{\boldsymbol{X}}}\xi ({R}_{i})).$$

Once force is determined, the acceleration of the particle is calculated from ${{\boldsymbol{f}}}_{i}^{{\rm{int}}}={m}_{i}{\ddot{{\boldsymbol{x}}}}_{i}$, where *m*_*i*_ is the mass of the particle.

Following our previous work^25,26^, we employed two numerical stabilization methods, noting further that different stabilization methods are commonly used in finite element solutions in continuum mechanics and transport. First, adding a penalty force to the particle forces (Eq. 9) that is reminiscent of an hourglass correction in the finite element framework, namely

10 $${{\boldsymbol{f}}}_{i}^{{\rm{hg}}}=\sum _{j\,\in S}\,-\,\alpha \,\frac{E{V}_{i}{V}_{j}\xi ({R}_{j})}{2{R}_{j}^{2}}({\delta }_{i}+{\delta }_{j})\frac{{{\boldsymbol{r}}}_{j}}{{r}_{j}},$$

penalizes non-zero modes of deformations due to rank-deficiency in SPH. Here, *E* is a stiffness-related penalty parameter and *α*∈[5, 50] is a control parameter determined via a sensitivity study^40^. The quantities *δ*_*i*_ and *δ*_*j*_ are length parameters that depend on the deformation gradients and relative positions of the particles, as explained in^40^.

Second, introducing a viscosity in the interaction of particles dissipates spurious vibrations of the particles, which is not meant to model the viscosity of interstitial fluid or cytoplasm, which obviously exist, but rather to render the dynamical solution stable. The first Piola-Kirchhoff stress tensor associated with viscous dissipation is defined as

11 $${{\boldsymbol{P}}}_{i}^{{\rm{visc}}}=2\eta {J}_{i}{{\boldsymbol{d}}}_{i}{{\boldsymbol{F}}}_{i}^{-T},$$

where ${{\boldsymbol{d}}}_{i}=({{\boldsymbol{l}}}_{i}+{{\boldsymbol{l}}}_{i}^{T})/2$ is commonly called the stretching tensor, with ${{\boldsymbol{l}}}_{i}={\dot{{\boldsymbol{F}}}}_{i}{{\boldsymbol{F}}}_{i}^{-1}$ the spatial velocity gradient and ${\dot{{\boldsymbol{F}}}}_{i}=({{\boldsymbol{F}}}_{i}^{t+1}-{{\boldsymbol{F}}}_{i}^{t})/\Delta t$ the rate of change of the deformation gradient between two time steps (*t* and *t* + 1) for a computational time interval Δ*t*. An appropriate value for the artificial viscosity *η* can be determined by trial and error; its final value does not affect the stress state when equilibrated, which is of most interest herein. Once the total force acting on a particle is calculated, we use a leapfrog time integration scheme to determine the position of the particle in the next time step and then repeat the process.

### Implementation of SPH for the aorta

#### Passive mechanical properties

Consistent with the primary microstructural constituents of the normal aortic wall, SPH particles are categorized into three main groups: particles *M*_*el*_ (red in Fig. 1) primarily model the elastic lamellae of the media, *M*_*int*_ (blue) primarily represent intra-lamellar SMCs, diffuse GAGs, and fibrillar collagen within the media, and *A* (green) represent the extracellular matrix of the adventitia, which consists primarily of collagen. The general form of strain energy function at each particle is^36^

12 $$W({{\boldsymbol{C}}}_{i},{{\boldsymbol{M}}}_{i}^{k})={\varphi }_{\Gamma }^{e}(\frac{\mu }{2}({I}_{1i}^{e}-3)-\mu \,\mathrm{ln}\,{J}_{i}+\frac{\hat{\lambda }}{2}{(\mathrm{ln}{J}_{i})}^{2})+\mathop{\sum }\limits_{k=1}^{4}\,{\varphi }_{\Gamma }^{{c}_{k}}(\frac{{c}_{1}^{k}}{4{c}_{2}^{k}}(\exp \,[{c}_{2}^{k}{({I}_{4i}^{k}-1)}^{2}]-1)).$$

The first term accounts for the elastin-dominated behavior, which is described by a modified neo-Hookean (isotropic) relation with *μ* a shear modulus and $\hat{\lambda }$ penalizing volume changes associated with particle motions. A nearly-incompressible behavior can be enforced by assuming a relatively large $\hat{\lambda }$ (e.g., $\hat{\lambda }/m$ = 1000 or larger), similar to its use in standard continuum calculations. ${J}_{i}=\det ({{\boldsymbol{F}}}_{i}^{2D}){\lambda }_{zi}$ is the remaining volume change, which accounts for area changes in two-dimensions (through $\det ({{\boldsymbol{F}}}_{i}^{2D})={F}_{i11}{F}_{i22}-{F}_{i12}{F}_{i21}$) multiplied by the axial stretch *λ*_*zi*_. Note, too, that ${I}_{1i}^{e}={\rm{tr}}({{\boldsymbol{C}}}_{i}^{{\boldsymbol{e}}})={\rm{tr}}({({{\boldsymbol{F}}}_{i}^{e})}^{T}{{\boldsymbol{F}}}_{i}^{e})$ is the first invariant of the right Cauchy-Green tensor and ***F***_*i*_^*e*^ is the deformation gradient tensor for an elastin-dominated particle (superscript *e*). Importantly, we assume a baseline neoHookean (isotropic) behavior of the thin elastic laminae, but introduce anisotropy with respect to the homeostatic configuration via different deposition stretches in the axial and circumferential directions (Table 1), which results in good fits to the biomechanical data. The second term represents the stored energy of the SMCs, diffuse GAGs, and collagen fibers, both medial and adventitial. SMCs are assumed to be oriented along the circumferential direction and their passive and active stresses act in this direction. Collagen fibers are divided into four families defined by locally oriented fiber bundles, namely axial (*k* = 1), circumferential (*k* = 2), and symmetric diagonal (*k* = 3, 4) families. For simplicity, the passive mechanical contribution of the SMCs is added to that of the circumferentially-oriented collagen family (*k* = 2). The exponential term in this stored energy function captures the strain-stiffening of the collagen matrix due to the straightening of the fibers with stretch. ${I}_{4i}^{k}$ is the square of the stretch associated with the *k*^*th*^ family, obtained by

13 $${I}_{4i}^{k}={{\boldsymbol{C}}}_{i}^{k}\,:{{\boldsymbol{M}}}^{k}\otimes {{\boldsymbol{M}}}^{k},$$

where ***M***^*k*^ = [0, sin *α*, cos *α*] is a unit vector associated with the direction of the *k*^th^ family, with *α* = 0, 90, ±*α*_0_ corresponding to axial, circumferential, and diagonal directions. To delineate the tensile and compressive (due to support by GAGs) stiffness of the collagen fibers and SMCs, we separate material parameters for tension (${c}_{1}^{k}={c}_{1}^{k+}$ and ${c}_{2}^{k}={c}_{2}^{k+}$ when ${I}_{4i}^{k}\ge 1$) and compression (${c}_{1}^{k}={c}_{1}^{k-}$ and ${c}_{2}^{k}={c}_{2}^{k-}$ when ${I}_{4i}^{k} < 1$).

**Table 1:** Model parameters for the multi-layered SPH model, some adapted from^58^.

Parameter	Symbol	Value
Inner and outer homeostatic radii, medial and adventitial thickness	${R}_{{\rm{in}}}$, ${R}_{{\rm{out}}}$ ${H}_{{\rm{M}}},{H}_{{\rm{A}}}$	646.8 μm, 687.0 μm, 28.4 μm, 11.8 μm
Elastin material constant	$\mu$	89.71 *kPa*
Medial axial and diagonal collagen material parameters (in tension + and compression -)	${c}_{1}^{k=1,3,4+}$, $\,{c}_{2}^{k=1,3,4+}$, $\,{\alpha }_{0}$ ${c}_{1}^{k=1,3,4-}$, $\,{c}_{2}^{k=1,3,4-}$	234.9 *kPa*, 4.08, 29.91° 29.14 *kPa*, 4.08
Medial SMC + circumferential collagen material parameters (+and −)	${c}_{1}^{k=2+}$, $\,{c}_{2}^{k=2+}$ ${c}_{1}^{k=2-}$, $\,{c}_{2}^{k=2-}$	261.4 *kPa*, 0.24 249.5 *kPa*, 0.15
Adventitial axial, circumferential, and diagonal collagen parameters (+ and −)	${c}_{1}^{k=1,2,3,4+}$, $\,{c}_{2}^{k=1,2,3,4+}$, $\,{\alpha }_{0}$ ${c}_{1}^{k=1,2,3,4-}$, $\,{c}_{2}^{k=1,2,3,4-}$	234.9 *kPa*, 4.08, 29.91° 29.14 *kPa*, 4.08
Medial elastin particle mass fractions	${\varphi }_{{M}_{e}}^{e},{\varphi }_{{M}_{e}}^{{c}_{1,2,3,4}}$	1.0, 0
Medial intra-lamellar particle mass fractions	${\varphi }_{{M}_{int}}^{e},{\varphi }_{{M}_{int}}^{{c}_{2}},{\varphi }_{{M}_{int}}^{{c}_{1,3,4}}$	0.1^#^, 0.84^#^, 0.06^#^
Medial intra-lamellar particle collagen orientations	${\beta }_{{M}_{int}}^{z},\,{\beta }_{{M}_{int}}^{d}$	0.056, 0.944
Adventitial particle mass fractions	${\varphi }_{A}^{e},{\varphi }_{A}^{{c}_{1,2,3,4}}$	0.04, 0.96
Adventitial collagen content	${\beta }_{A}^{z},\,{\beta }_{A}^{d},{\beta }_{A}^{\theta }$	0.067, 0.877, 0.056
Elastin deposition pre-stretch	${G}_{\Gamma ,r}^{e},{G}_{\Gamma ,\theta }^{e},{G}_{\Gamma ,z}^{e}$	0.32, 1.90, 1.62
Medial SMC + circumferential collagen deposition pre-stretch	${G}_{{M}_{int}}^{k=2}$	1.20
Other medial and adventitial collagen deposition pre-stretch	${G}_{{M}_{int}}^{k=1,3,4},{G}_{A}^{k=1,2,3,4}$	1.25
Limiting circumferential stretches for active stress	${\lambda }_{{\rm{\max }}}$ ${\lambda }_{{\rm{\min }}}$	1.1^#^ 0.6^#^
Applied stimulus of the active tone	${T}_{\max }$	500^#^ *kPa*
Shear modulus of the GAG particles	${\mu }^{{\rm{GAG}}}$	0.1 *kPa*

# denotes that the parameter was determined by fitting model results to experimental data for pressure and outer diameter for the descending thoracic aorta of mouse^39,43^.

**Figure 2:**
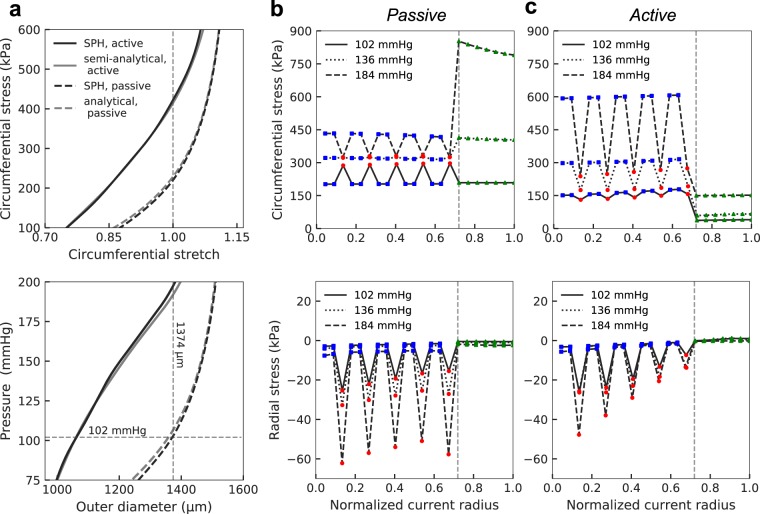
Predicted wall stress with explicit inclusion of elastic lamellae and intra-lamellar regions. (**a)** The bulk behavior of the aorta predicted by passive (dashed black line) and active (solid, black line) SPH models agrees well with an analytical passive solution for a homogenized media (dashed, grey line) and a semi-analytical active solution determined by adding active stress to the passive SPH result (grey, solid line). Vertical and horizontal dashed lines show the homeostatic *in vivo* configuration. Note the 23% reduction in outer diameter at the homeostatic pressure (102 mmHg) caused by SMC activation. **(b**,**c**) Transmural distributions of circumferential and radial Cauchy stress through the thickness in the passive and active models, respectively. The red circle, blue square, and green triangle correspond to medial elastin, medial intra-lamellar, and adventitial particles, respectively; the vertical dashed line shows the medial-adventitial border in the current configuration. The highly non-uniform distribution of stress through the wall is attributed to different properties of the elastic lamellae and intra-lamellar regions. Active stress generation by SMCs elevates stress in the intra-lamellar particles but, because of the reduction in the diameter, decreases stresses in the medial elastin and adventitial particles relative to those in the passive case. Note that the assumed deposition stretches tend to reduce transmural gradients, especially in the homeostatic state, by introducing residual stresses (not shown).

Following our previous work on the mouse aorta^41^, mass fractions for elastin and collagen in the adventitia were ${\varphi }_{A}^{e}=0.04,\,{\varphi }_{A}^{{c}_{1,2,3,4}}=0.96$. Given that our prior model was based on a uniform, homogenized media, the associated mass fractions had to be adjusted to account for heterogenity in the current model. The mass fraction of SMCs and collagen were set to zero (${\varphi }_{{M}_{e}}^{{c}_{1,2,3,4}}=0$) for particles that represent elastic lamellae, thus these particles only exhibit a neo-Hookean behavior (${\varphi }_{{M}_{e}}^{e}=1$). The mass fractions for the intra-lamellar particles ${\varphi }_{{M}_{int}}^{{c}_{k}}$ were determined by fitting experimental pressure-diameter data^39^, which simultaneously yielded an overall validation by describing associated circumferential stress-stretch data. Results of such fitting are shown in Fig. 2a. Although the SMCs and medial collagen fibers are oriented primarily within the circumferential-axial plane, the associated particles were also endowed with radial stiffness (to represent radial elastic fibers and GAG-supported collagen in compression) via a small fraction of neo-Hookean matrix (with ${\varphi }_{{M}_{int}}^{e}=0.1$). Finally, to distinguish between the collagen fibers in axial (superscript *z*), circumferential (superscript *θ*), and diagonal (superscript *d*) directions, we multiply ${\varphi }_{{M}_{int}}^{{c}_{1,3,4}},{\varphi }_{A}^{{c}_{1,2,3,4}}$ by appropriate fractions ${\beta }_{{M}_{int},A}^{z},{\beta }_{{M}_{int},A}^{d},{\beta }_{A}^{\theta }$. Values of the parameters are listed in Table 1.

Different from many aortic studies, we use the *in vivo* homeostatic configuration as our reference configuration^36^, thus avoiding the need to model constitutive behaviors at unphysiologically low pressures and axial loads or the need to capture large particle motions associated with loading from a traction-free to the *in vivo* state. The deformation gradient ***F***_*i*_ and right Cauchy-Green ***C***_*i*_ tensors equal identify at the passive homeostatic reference configuration (i.e., ***F***_*i*_ = ***I*** and ***C***_***i***_ = **(*F***_***i***_**)**^***T***^***F***_***i***_ = ***I***). To capture the homeostatic stress in this loaded configuration, we introduce “deposition pre-stretches” for each constituent, consistent with constrained mixture formulations^36^. The deposition pre-stretch tensor for elastin is denoted by ${{\bf{G}}}_{\Gamma i}^{e}$ and deposition pre-stretches for the SMCs and collagen fiber families “*k*” are denoted by ${{\rm{G}}}_{\Gamma i}^{k}$, where (Γ = *M*_*el*_, *M*_*int*_, *A*), denote medial elastin, medial intra-lamellar, and adventitial particles, respectively. These deposition pre-stretches are introduced in the reference configuration under plane strain (i.e., at a fixed axial stretch, *λ*_*zi*_ = 1) and at a fixed inner radius^36^. Given the associated mean circumferential wall stress *σ*_*θ*_, current inner radius *r*_in_, and wall thickness *h*, one can then calculate the distending pressure *P* and mean circumferential stretch *λ*_*θ*_ in the homeostatic reference configuration:

14a $$P=\frac{{\sigma }_{\theta }h}{{r}_{{\rm{in}}}},$$

14b $${\lambda }_{\theta }=\frac{{r}_{{\rm{in}}}+h/2}{{R}_{{\rm{in}}}+H/2}.$$

After achieving the homeostatic pre-stretched configuration, then consistent with a semi-inverse approach in nonlinear continuum mechanics we apply displacement boundary conditions on particles located at the inner radius of the wall to inflate/deflate the vessel to various non-homeostatic configurations. The deformation gradient of elastin used in Eq. 12 (${{\boldsymbol{F}}}_{i}^{e}$) and the right Cauchy-Green tensor of fiber family “*k*” used in Eq. 13 (${{\boldsymbol{C}}}_{i}^{k}$) are thus obtained by ${{\boldsymbol{F}}}_{i}^{e}={{\boldsymbol{F}}}_{i}{{\boldsymbol{G}}}_{\Gamma i}^{e}$ and ${{\boldsymbol{C}}}_{i}^{k}={({G}_{\Gamma i}^{k})}^{2}{{\boldsymbol{C}}}_{i}$, respectively.

#### Active biomechanical properties

The active stress generated by SMC contraction is assumed to act primarily in the circumferential direction. Following Rachev and Hayashi^41,42^, we introduced the Cauchy stress due to active tone as an additional term to the circumferential stress of the intra-lamellar particle *i*, namely

15 $${\sigma }_{\theta ,i}^{{\rm{act}}}={\varphi }_{{M}_{int}}^{{c}_{2}}\,{T}_{{\rm{\max }}}\,{\lambda }_{\theta ,i}[1-{(\frac{{\lambda }_{{\rm{\max }}}-{\lambda }_{\theta ,i}}{{\lambda }_{{\rm{\max }}}-{\lambda }_{{\rm{\min }}}})}^{2}],$$

where *λ*_*θ*,*i*_ is the circumferential stretch of particle *i* with respect to the reference configuration of the passive model. This active stress reaches its maximum value when circumferential stretch *λ*_*θ*,*i*_ equals *λ*_max_ and it vanishes at *λ*_min_. The parameter *T*_max_ regulates the maximum active stress depending on the applied stimulus, including calcium influx. *T*_max_,*λ*_max_, and *λ*_min_ are specified such that SMC contraction at the homeostatic pressure (102 mmHg) reduces the outer diameter by 20–25%, in agreement with measurements for the murine descending thoracic aorta^43^.

#### Disruption of the elastic lamellae

Having particles represent individual elastic lamellae allows us to model their disruption and the subsequent redistribution of stress to other constituents without introducing an additional constitutive model for the damage process. Rather, this lamellar damage is accomplished numerically via the following steps: (i) a segment within an elastic lamella is selected and the strain energy of the associated particles is reduced to zero by decreasing to zero their material parameters in the neo-Hookean strain energy function (*μ*, $\hat{\lambda }$), (ii) a thorough search of the list of neighboring particles allows ruptured lamellar particles to be removed from the lists, and (iii) the non-damaged particles belonging to the same elastic lamella but located on either side of the disrupted segment are also removed from the neighborhood lists of the particles located on the opposite side. This final step captures the severance of the elastic lamella via complete disconnection of the particles located on each side of the disrupted segment. Depending on the purpose of the simulation, one or more individual elastic lamellae can rupture but these computational steps are performed independently at each lamella. Finally, a relaxation step dissipates possible oscillations that arise naturally when severing inter-connected elastically stretched particles, thus resulting in a final converged equilibrium solution.

#### Replacement of SMCs by GAGs

To model the loss of SMC function, then apoptosis and replacement with GAGs, a pool of intra-lamellar particles is assumed to experience the following three steps:

1. *Loss of contractility:* First, the active stress of the designated particles is reduced to zero while the remaining intra-lamellar SMC particles maintain their active stress.
2. *Reduction in stiffness:* Next, the material behavior of the designated medial particles transforms to that of a pure GAG particle (orange in Fig. 1). Specifically, these particles lose their original passive stiffness (due to the smooth muscle and intramural elastic and collagen fibers) and, instead, adopt a modified neo-Hookean stress function to model the isotropic behavior of a GAG pool that fills the space left void by the apoptotic cells^44^. This modified neo-Hookean behavior also captures the compressive resistance associated with the GAGs and their possible interactions with intramural collagen fibers^12^. For a GAG pool particle labelled by *i*, with left Cauchy-Green tensor ${{\boldsymbol{B}}}_{i}={{\boldsymbol{F}}}_{i}{{\boldsymbol{F}}}_{i}^{T}$, the associated Cauchy stress is:
  16 $${{\boldsymbol{\sigma }}}_{i}^{{\rm{GAG}}}=\frac{1}{{J}_{i}}\,{\mu }^{{\rm{GAG}}}({{\boldsymbol{B}}}_{{\boldsymbol{i}}}-{\boldsymbol{I}}),$$
  noting that *J*_*i*_ is the volume change of the GAG particle and parameter values (*μ*^GAG^ = 0.1 kPa in^44^) assure that the pool is much less stiff in tension than are the medial elastin particles (*μ* = 89.7 kPa).
3. *GAG swelling:* Finally, we introduce a hydrostatic pressure that is generated by swelling of the negatively charged GAGs. By assuming a dilute concentration of GAGs within the interstitial fluid, a Gibbs-Donnan swelling pressure is added to the Cauchy stress function of Eq. 16:

17 $${{\boldsymbol{\sigma }}}_{i}^{{\rm{GAG}}}=\frac{1}{{J}_{i}}{\mu }^{{\rm{GAG}}}({{\boldsymbol{B}}}_{{\boldsymbol{i}}}-{\boldsymbol{I}})-RT(\sqrt{{({c}^{{\rm{FC}}})}^{2}+{({c}^{\ast })}^{2}}-{c}^{\ast }){\boldsymbol{I}},$$

where *R* and *T* are the universal gas constant and the absolute body temperature (310 °K), c^*^ is the ionic concentration of the surrounding medium (c^*^ = 300 mEq/l from^45^), and *c*^FC^ is the concentration of GAGs (with c^*^ = 200 mEq/l, equivalent to ~155 kPa Gibbs-Donnan swelling pressure^44^). GAG-associated swelling pressures effectively contribute to the compressive stiffness as well.

These three steps are performed for a pool of intra-lamellar particles located between two adjacent elastic lamellae (shown in Fig. 1) with various extensions in the circumferential direction. Of course, disruption of the elastic lamellae can be combined with these three steps and, for instance, can precede the loss of the contractility or follow the GAG swelling.

### Aortic model evaluation

#### Comparison to vessel-level data

As noted earlier, we previously used standard analytical and numerical solutions to verify and validate our extended SPH approach for nonlinear biosolids, including damage mechanics, and we validated predictions of bulk properties for a homogenized aortic wall model^25,26^. We similarly found herein that our new multi-lamellar aortic wall model described bulk murine aortic behaviors under both passive and active conditions, namely axially isometric pressure-diameter responses at pressures near homeostatic and axially isometric and isobaric diameter reductions in response to maximal smooth muscle contraction (Supplemental Fig. S1). Unfortunately, there are no lamellar-scale biomechanical data available for the mouse to enable validation of our individual lamellar unit model.

#### Particle distributions

To examine the dependence of simulations of pointwise wall stress on the original distribution of particles, we performed two comparisons of results for three different densities of particles. Specifically, recalling that the homeostatic wall thickness *H* = 40.2 μm, we varied particle density from 0.24 to 0.39 and then 0.62 particles per *μm*^2^ (effectively 2, 3, and 4 particles within each intra-lamellar space) and compared stress fields through the wall of a healthy vessel as well as in the vicinity of a simulated pool of GAGs with disrupted elastic lamellae (Supplemental Fig. S2). Results were nearly identical for these three densities for the healthy aorta. Results for the case of an intramural defect are more difficult to interpret because one cannot prescribe precisely the same size defect when using different particle densities. Nevertheless, results for the computed stress concentrations differed by only 4–6% for the three particle densities and particular intra-lamellar defects examined. Of course, we could not compare these lamellar results directly with simulations via other models because of the novel features of our current model. It should be noted that, due to the possible singularity of the stress at the “tip” of the disrupted elastic lamellae, the SPH results for those stresses are likely not fully reliable. In our analysis, however, we focus on stress concentrations along radial lines passing through the wall at the midpoint of the disrupted regions, hence examining radial load transferal. Such lines are shown in Fig. S2. Based on these simulations, we concluded that a density 0.24 particles per *μm*^2^ was sufficient, particularly given that length scales of interest (cell diameters, laminae thickness, and intra-lamellar distances) are of order 2–5 μm and above. This density is used in all simulations presented below.

## Results

### Normal values of wall stress

Consider, first, states of aortic wall stress under normal passive and active conditions at different luminal pressures. SPH predictions of bulk responses of the aorta, such as relations between luminal pressure and outer diameter as well as mean circumferential stress and stretch, are shown in Fig. 2a. For comparison, we also show an analytical solution for a passive wall, obtained by assuming a thin homogeneous vessel^41^, and a semi-analytical solution for a contracted wall, obtained by adding an active stress (Eq. 15) to the SPH result for the passive behavior (i.e. dashed black line in Fig. 2a). Such comparisons show that the multi-layered SPH model captures bulk behaviors well while including separate lamellar units that have been ignored in analytical solutions. Figure 2a also shows that SMC contraction constricts the vessel by ~23% (from *R*_out_ = 688 μm to *R*_out_ = 527 μm) in agreement with experimental findings for the murine descending thoracic aorta^43^.

Transmural distributions of stress predicted by the passive and active SPH models are shown in Fig. 2b,c, respectively. As expected, the presence of separate (~2 μm thick) elastic lamellae and associated intra-lamellar regions results in a highly non-uniform distribution of circumferential stress through the thickness. In the passive model (Fig. 2b, top), an increase in pressure from 102 mmHg to 184 mmHg causes the tissue within the intra-lamellar spaces (representing SMCs and elastic and collagen fibers) and adventitia (mostly collagen) to straighten and thereby increase the circumferential stress (Fig. 2b, top). At high pressures, the increase in adventitial stress is greater than the increase in medial stress, consistent with the adventitia acting as a protective sheath for the more vulnerable SMCs and elastic lamellae at elevated pressures^36^. Conversely, SMC contractions allow these cells to support greater circumferential stress than the elastic-lamellae and adventitia (Fig. 2c, top). Because this SMC contraction reduces the aortic diameter at a fixed pressure, the reduced stretches experienced by passive constituents (matrix) in the media and adventitia lowers the overall circumferential stress. Thickening of the wall at reduced diameters creates a slight transmural gradient in stress. For example, the circumferential stress at the elastin particles (red circles in Fig. 2c, top) increases from the inner to the outer radius at the homeostatic pressure of the active model (102 mmHg). The radial stress also distributes nonuniformly, with regions of negligible stress (within intra-lamellar spaces denoted by blue squares) and regions with larger compressive stress (corresponding to the elastic lamellae, red circles).

### Stress concentration induced by disrupted elastic lamellae

**Figure 3:**
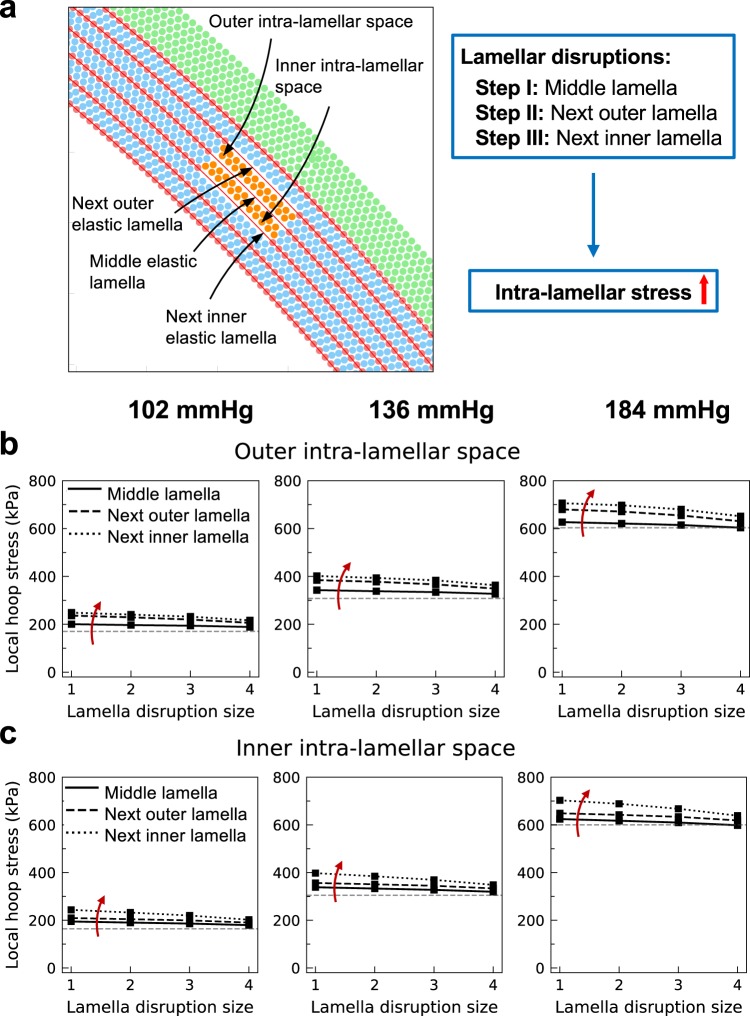
Stress increases in intra-lamellar regions due to disrupted medial elastic lamellae. (**a)** Three elastic lamellae, denoted middle, next outer, and next inner, are ruptured sequentially (steps I, II, and III; see red arrows), which increases the maximum circumferential stress at the outer **(b)** and inner **(c)** intra-lamellar particles. Horizontal dashed lines show the stress before disruption. Results are shown for 1–4 extents of disruption (angular sectors of 2°, 4°, 8°, and 20°; abscissa) and 3 luminal pressures (left-to-right). These increased stresses suggest that lamellar disruption can lead to damage of nearby SMCs, especially at higher pressures. Because rupture of the middle lamella alone does not induce a substantial increase in stress, remaining preserved lamellae may protect the intra-lamellar space against damage. Note that stresses are plotted transmurally, across the defect and not at the “tip” of the damaged region.

Consider, next, the effects on the wall due to disruption of elastic lamellae, particularly changes in circumferential stress experienced by the SMCs. All results were evaluated at three distending pressures (*P* = 102, 136, and 184 mmHg), but the same fixed axial stretch. Three elastic lamellae located at *R* = *R*_in_ + 0.4*H* = 663 μm (denoted as the middle elastic lamella in Fig. 3a), *R* = *R*_in_ + 0.27*H* = 657 μm (next inner elastic lamella), and *R* = *R*_in_ + 0.54*H* = 668 μm (next outer elastic lamella) were sequentially disrupted, with the extent of disruption compared for four cases (angular zones of 2°, 4°, 8°, and 20°). Following the first rupture of an elastic lamella (middle lamellar disruption), the circumferential stress increased within the inner and outer intra-lamellar domains, though not significantly when compared to baseline values (Fig. 3b,c). With further disruption of the next two lamellae, the stress of the same intra-lamellar particles increased further (Fig. 3b,c, next outer and inner lamellae rupture). This increase in stress is higher at elevated pressures since the elastic lamellae carry a higher circumferential stress at elevated pressures and thus transfer a larger load to the adjacent intra-lamellar space upon disruption (compare ~50 kPa increase in stress at 102 mmHg with ~100 kPa at 184 mmHg). The elevation in stress was less for larger disrupted regions, however, consistent with stress concentrations being greater when the region of degeneration is smaller. It is thus expected that a full circumferential disruption would eliminate the local circumferential stress concentration at the nearby inner and outer edges of the damaged area, thus yielding a more uniform stress circumferentially. Altogether, these results suggest possible roles of lamellar damage, including excessive stress in the radially adjacent intra-lamellar regions that can initiate damage of intra-lamellar SMCs. Notably, while disruption of a single lamella can induce damage in the SMCs, intact close-by lamellae can protect the enclosed intra-lamellar space; thus, rupture of additional lamellae may be needed to induce SMC damage.

### Stress concentration induced by GAG replacement of SMCs

**Figure 4:**
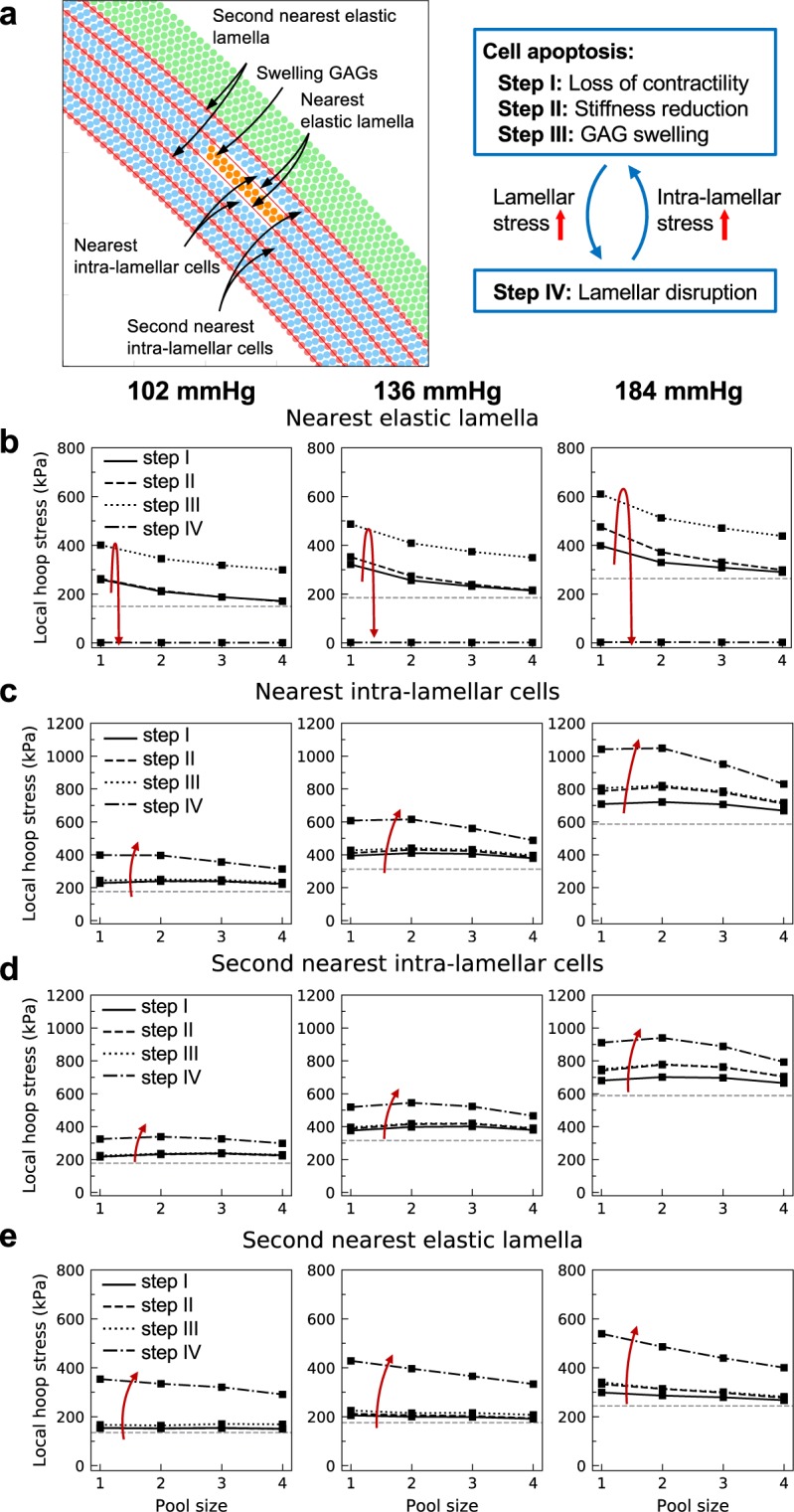
Stress concentrations near a pool of cells undergoing apoptosis and replacement with GAGs. (**a)** Pools of intra-lamellar particles defined by four angular extents (2°, 4°, 8°, and 20°) are assumed to lose contractility and matrix stiffness (step I), be replaced with GAGs having reduced tensile stiffness (step II), and gain Gibbs-Donnan type swelling (step III; see red arrows for sequence), thus changing the maximum circumferential stress in the **(b)** nearest elastic lamella, **(c)** nearest intra-lamellar space, **(d)** second nearest intra-lamellar space, and **(e)** second nearest elastic lamella (all shown in **(a)**). The horizontal dashed lines show the baseline stress before disease. Loss of contractility and reduction of stiffness (steps I and II) create a stress concentration in the nearby particles that increases with distending pressure (left-to-right) but decreases with the size of the pool. GAG swelling (step III) increases the stress in the nearest elastic lamella while not affecting distant locations. Once the nearest elastic lamellae rupture (step IV, lamellar disruption), stress in the intra-lamellar regions increases dramatically **(c**,**d)**, suggesting a greater chance of further SMC damage in these areas. Also note the jump in stress in the second nearest elastic lamella following disruption of the nearest elastic lamella **(e)**, with damage within an intra-lamellar space propagating radially. Overall, these results suggest a feedback loop between cell apoptosis and lamellar disruption, which, independent of the initiating event, can lead to catastrophic damage within the wall (shown in **(a)**).

Four pools of intra-lamellar particles with angular sectors of 2°, 4°, 8°, and 20° experienced three steps of SMC dysfunction, apoptosis, loss of medial collagen, and replacement with GAGs, as defined in section 2.2.4. Of particular interest is the change in circumferential stress in the vicinity of a pool, particularly within the nearest and second nearest lamellar and intra-lamellar regions (shown in Fig. 4a). During the first step, loss of contractility within the region increased the circumferential stress in nearby regions (Fig. 4b,c, step I: loss of contractility). As SMCs apoptosed and were replaced by GAGs, the reduced tensile stiffness led to an increased circumferential stress experienced by the particles (Fig. 4b,c, step II: stiffness reduction). At lower pressure (*P* = 102 mmHg in Fig. 4b,c), the passive stress of the cells was low and consequently the reduction in stiffness did not cause a noticeable change in stress. On the other hand, reducing the stiffness of the region/pool at higher pressures (*P* = 136 or 184 mmHg) caused a marked increase in circumferential stress in surrounding regions. As expected, this increase in stress was stronger at the closest particles and decayed at the second-nearest ones (compare with Fig. 4d,e). The model also suggests that the stress concentration decreases with the size of the pool, thus smaller lamellar disruptions tend to create higher stress concentrations. Similar to the size effect of lamellar disruption (section 3.2), this finding is consistent with the expectation that a pool that extends around the full circumference will not induce a circumferential stress concentration. That is, localized, not distributed, defects result in greater stress concentrations.

Swelling GAGs within a pool resulted in a much higher circumferential stress on the elastic lamellae around the pool (Fig. 4b, step III: GAG swelling), but, interestingly, with only modest effects on stress in the nearest intra-lamellar space (Fig. 4c, step III). This result underscores a protective role of elastic lamellae against alterations within their intra-lamellar regions. We then added one additional step (step IV: lamellar disruption) and assumed that the increase in stress in the nearest elastic lamellae caused lamellar rupture. The stress in the disrupted elastic lamellae dropped to zero (Fig. 4b, step IV), and the excessive stress transferred to the adjacent intra-lamellar space, substantially increasing circumferential stress in the nearest (Fig. 4c) and second-nearest (Fig. 4d) intra-lamellar regions. This result demonstrates how SMC apoptosis and loss of medial matrix, with replacement with GAGs that can swell in one layer, can induce damage in SMCs and matrix in neighboring intra-lamellar regions through rupture of the shared elastic lamella. Interestingly, following rupture of the nearest elastic lamellae, there was a marked increase in stress in the second nearest elastic lamellae (Fig. 4e, step IV), a process that could repeat. This finding suggests that swelling within an intra-lamellar space with subsequent lamellar disruption increases the vulnerability of distant elastic lamellae to rupture and reveals a mechanical mechanism that can explain a transmural propagation of damage. Moreover, these results suggest a positive feedback loop between cell apoptosis, loss of matrix, and replacement with GAGs and the disruption of adjacent elastic lamellae. Independent of the triggering event within such a feedback loop, cell apoptosis can induce lamellar disruption, which in return can increase the stress experienced by other SMCs and lead to further cell apoptosis (Fig. 4a).

### Stress concentration induced by two pools of apoptotic SMCs and GAGs

**Figure 5:**
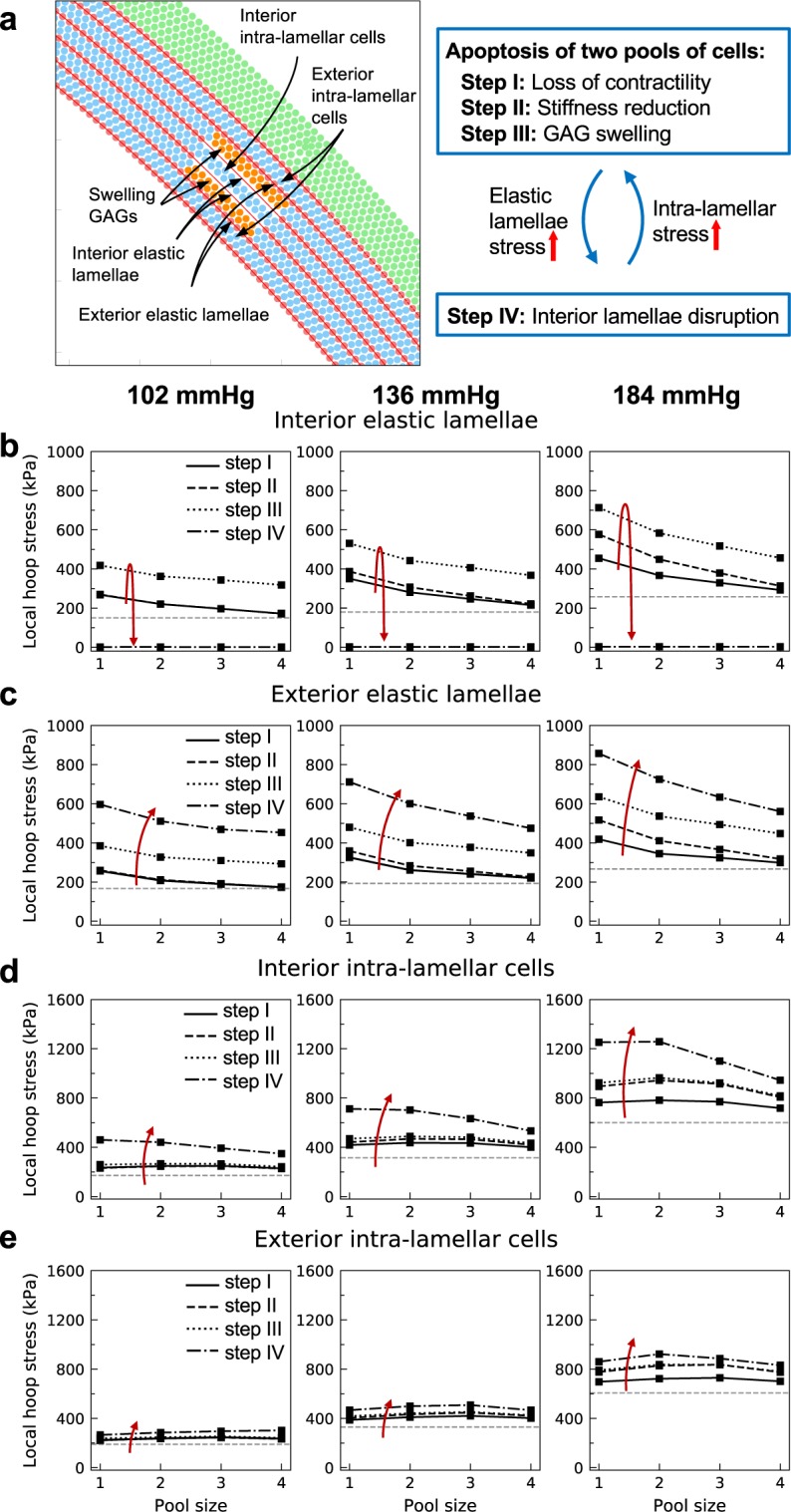
Feedback loop caused by apoptosis of two localized collections of cells separated by an intact intra-lamellar space but with elastic lamellar disruption. (**a)** Two groups of cells with identical angular extents (2°, 4°, 8°, and 20°, referred to as pool size 1–4) are assumed to lose contractility, reduce in stiffness, and be replaced with GAGs (steps I, II, and III; red arrows). Following cell apoptosis/loss of intra-lamellar matrix and replacement with GAG (steps I-III), the **(b)** interior elastic lamella experiences a higher rise in stress compared with the **(c)** exterior elastic lamella, especially at higher pressure. Following disruption of the interior elastic lamellae (step IV, lamellar rupture), the stress in the interior intra-lamellar space **(d)** increases more drastically compared with the exterior intra-lamellar space **(e)**, suggesting a high vulnerability of SMCs in the region surrounded by apoptotic pools of cells and GAGs to damage. Baseline levels of stress are again shown by horizontal dashed grey lines.

Consider, next, possible effects of two regions of SMCs undergoing apoptosis with associated loss of matrix and replacement by GAGs. Two pools were assumed to have equal angular extents but separated by an intact intra-lamellar space. Comparing the stress concentration within the space between the two pools (Fig. 5a) with that caused by a single pool (i.e., comparing Figs 5b with 4b) shows that at lower pressures (102 and 136 mmHg), the elastic lamellae located between the two pools experienced a similar or slightly larger stress compared with the elastic lamellae around a single pool (410 kPa vs. 400 kPa at 102 mmHg). This result reveals a strong locality of stress concentrations near such pools, presumably due to the overall wall thickness and the larger distance between the pools at low pressures. On the other hand, at a higher pressure (184 mmHg), the stress experienced by the interior elastic lamellae between the two pools was greater than that exerted on the elastic lamellae surrounding a single pool (710 kPa vs. 610 kPa at 184 mmHg), suggesting that at higher pressures, which thins the wall, multiple pools can collectively increase wall stresses. The model also shows a higher stress at the interior elastic lamellae (Fig. 5b) compared with that in the exterior lamellae (Fig. 5c), though this difference is negligible at lower pressures. With further disruption of the interior elastic lamellae, the increase in stress in the enclosed intra-lamellar cells and matrix (Fig. 5d, step IV: lamellar disruption) is more pronounced than for cells near a single pool (Fig. 4c, step IV), especially at higher pressures. As expected, following lamellar disruption, the SMCs and matrix located at the exterior intra-lamellar regions (Fig. 5e) also experienced an increase in stress, though this increase was modest because they were located away from the damaged region. This finding suggests a higher chance of SMC damage for cells located between two degenerate pools. Note, too, that upon disruption of the interior elastic lamellae, there was a substantial rise in the stress in the exterior elastic lamellae (Fig. 5c, step IV), which could initiate a sequential rupture of elastic lamellae and perhaps transmural damage propagation.

**Figure 6:**
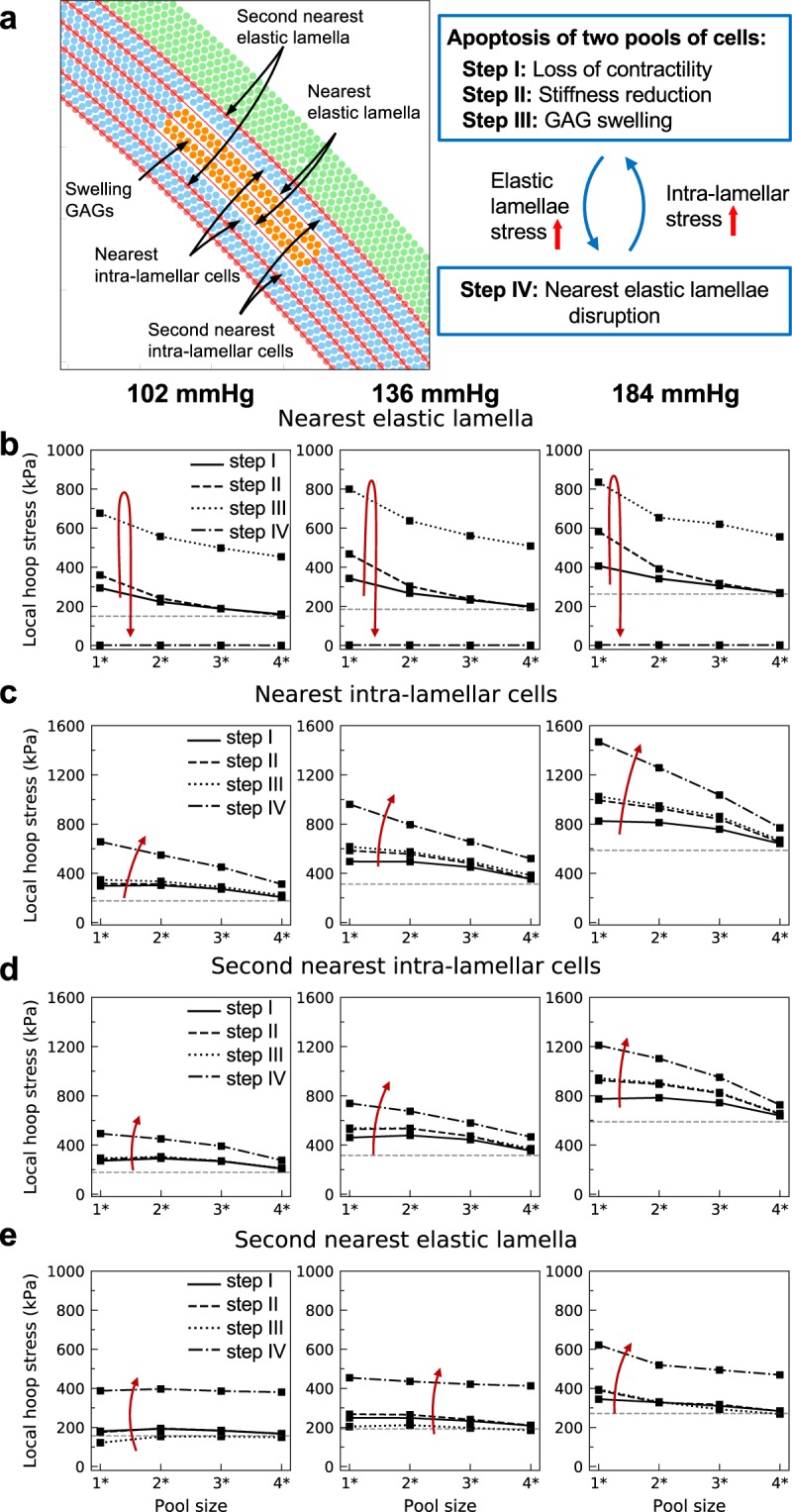
Stress concentration near two merged pools of cells undergoing apoptosis and replacement with GAGs. (**a)** Two collections of cells belonging to neighboring intra-lamellar regions are replaced by GAGs. The elastic lamella separating the two pools was removed prior to the study and the angular extent of the pools was assumed to be twice that of the single pool studied in Fig. 4 (1* = 4°, 2* = 8°, 3* = 16°, 4* = 40°). Shown are changes in circumferential stress of the **(b)** nearest elastic lamella and **(c)** intra-lamella space, as well as the second nearest **(d)** intra-lamellar space and **(e)** elastic lamella. Horizontal dashed lines show the baseline stress before introducing the pools. The model predicts that stress concentrations caused by two merged pools are in general higher than for a single-pool situation, especially at higher pressures. Rupture of the nearest elastic lamellae allows the elevated stress to reach the nearest and second nearest intra-lamellar cells (**c** and **d**), and to increase the chance of SMC damage. Meanwhile, the stress in the second nearest elastic lamellae also experiences a jump (**e**, step IV: lamellar disruption), more than the case with a single pool (Fig. 4e), thus predicting facilitated lamellar disruption and propagation of damage into the wall.

If one further assumes that the large stress generated due to ruptured elastic lamella around a single pool damages the cells/matrix in the nearby intra-lamellar space, this could drive them to become apoptotic/degraded and be replaced with GAGs. Hence in Fig. 6a, two collections of intra-lamellar particles, this time merged by the removal of the separating elastic lamella, were assumed to be lost (steps I-III). Because the stress caused by multiple swelling pools was higher than that caused by a single pool, we increased the sectors of the pools in the circumferential direction from (2°, 4°, 8°, and 20°) to (4°, 8°, 16°, and 40°), knowing that increasing the pool size lowers the stress concentrations. Interestingly, this simulation suggests a growth of pools in the circumferential direction via delamination of the intra-lamellar space at the tip of the pools, a general result that was found previously though without delineation of separate medial constituents^25^.

Comparing the circumferential stress induced by two merged pools (Fig. 6b–e) with that for a single pool (Fig. 4b–e) suggests a more drastic elevation of stress in the former, especially at higher pressures, even though the angular sectors of the pools is double that in the two-pooled system. As apoptotic cells and lost matrix were replaced by GAGs (i.e., following loss of contractility, reduction in stiffness, and GAG swelling, steps I-III), the stress increased in the nearest and second-nearest elastic lamellae (Fig. 6b,e) and within the intra-lamellar spaces (Fig. 6c,d). An exception arose for the stress in the second nearest elastic lamella upon GAG swelling (Fig. 6e, step III: GAG swelling). In this case, because of the incompressibility of the elastic lamellae, the compressive radial stress caused by the swelling GAGs facilitated the extension of the elastic lamellae in the circumferential direction and lowered its tensile circumferential stress. Nonetheless, in all cases, the circumferential stress increased upon disruption of the nearest elastic lamellae (Fig. 6c–e, step IV: lamellar disruption). It should also be noted that the elastic lamella separating the two pools in the baseline model had already been disrupted prior to these simulations, thus in this case the resistance generated from the unbroken lamellae was compromised. Finally, following lamellar rupture, the final stress in the second elastic lamella was higher compared with that for the single pool (Fig. 5e, step IV compared with Fig. 4e). Overall these observations suggest that spreading of GAGs from one intra-lamellar region into neighboring intra-lamellar spaces increases the vulnerability of the distant elastic lamellae to disruption and, hence, damage produced by multiple pools can propagate catastrophically through the wall.

**Figure 7:**
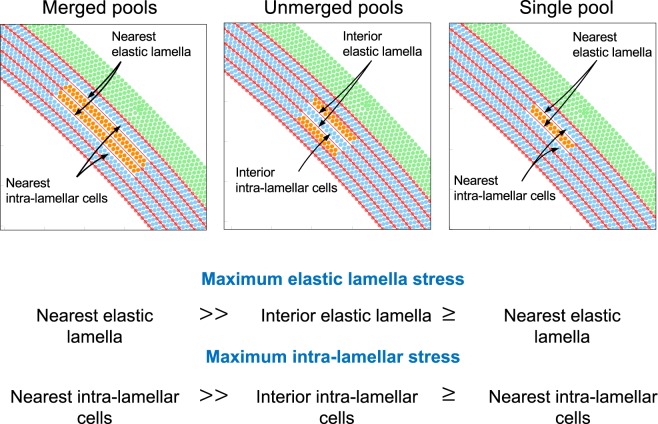
Comparison of stress fields induced by a single pool, two unmerged pools, or two merged pools of apoptotic cells replaced with GAGs. Overall, the stresses acting on elastic lamellae and intra-lamellar cells near two merged-pools is the highest, indicating a higher chance of lamellar disruption and SMC dysfunction in this case. Note that (≥) indicates that the stress concentrations near two unmerged pools and a single pool are relatively close at low pressures (102 mmHg), but the two unmerged pools create a higher stress in their surroundings at higher pressure (184 mmHg).

In summary, stress concentrations generated by a single pool (Fig. 4), two unmerged pools (Fig. 5), and two merged pools (Fig. 6) of apoptotic cells/lost matrix are compared in Fig. 7. The elastic lamellae and the intra-lamellar regions near multiple merged pools experienced the largest increase stress and therefore were more prone to failure and dysfunction. Comparing stress concentrations for the single pool and the two unmerged pools shows that stress concentrations are highly localized around pools at lower pressures, but the effects of multiple pools become more collective at higher pressures. Overall, these results suggest that extension of SMC dysfunction and associated losses of matrix from an intra-lamellar space into an adjacent intra-lamellar space affects stress concentrations and can lead to catastrophic damage.

## Discussion

Notwithstanding the considerable detail available on the microstructural architecture of the aortic media^3,29–31^, most models of aortic wall mechanics in health and with aneurysmal dilatation (e.g.^46–49^) as well as for aortic dissection (e.g.^44,50,51^) have relied on standard methods of continuum biomechanics and locally homogenized material properties. Much has been learned via such approaches, but there is clearly a need to consider greater microstructural detail. In contrast, among others, Thunes *et al*.^22,52^ proposed a microstructural model of a medial lamellar unit, including dense networks of fibers that separately represent elastic lamellae and intra-lamellar collagen. This model was specialized for an ideal rectangular geometry relevant to *in vitro* mechanical testing, and hence did not include residual stresses, SMCs or their ability to vasoconstrict the wall, or an adventitial layer that can stress-shield the media.

Herein, we adopted a particle-based approach enriched to include a murine-specific aortic (cylindrical) geometry, physiological loads, residual stress (arising from prescribed deposition stretches in the media and adventitia), individual lamellar structures consisting of different structural proteins, SMC contractility and apoptosis, GAG accumulation and swelling, and subsequent intramural damage, thus building on but significantly extending our prior SPH work. Specifically, we previously developed and implemented a SPH model to examine stresses around a pool of swelling GAGs within an otherwise homogenized model of the aortic media^25^. This model confirmed that GAGs can increase the radial stresses beyond a certain threshold and possibly cause intra-lamellar delamination. The model also revealed that damage within the media can allow GAG pools to grow circumferentially and even to coalesce with other pools. Yet, given that the media was modeled as a homogenized layer, we could not examine biophysical mechanisms related to the disruption of individual elastic lamella that separate GAG pools in local regions. Hence, the primary novelty of the current model is our delineation of medial particles associated with both elastic lamellae and intra-lamellar constituents, including SMCs that can contract, relax, or drop-out and GAGs that can pool and swell. Comparison of our prior and present results suggest, for the first time, that SMC dysfunction and apoptosis and the associated loss of extracellular matrix constituents combined with a local accumulation of GAGs can markedly increase the circumferential stress in the elastic laminae and initiate a repetitive rupture of the lamellar structures in the radial direction. Notably, combined propagation of a dissection in circumferential and radial directions is common, leading to extensive lesions^1–3^. Importantly, it is increasingly appreciated that SMC phenotypic modulation and/or drop-out^53,54^ and localized pooling of GAGs^12,13^ can play important roles in aortic dissection. Indeed, even just loss of SMC contractility appears to be fundamental to disease development or progression^34,55^. Of course, it has long been known that loss of elastic fiber integrity is key in cases such as Marfan Syndrome^53,54^. Our model confirms such effects quantitatively and shows for the first time how these separate contributors can also act collectively.

Specifically, our model suggests that highly localized defects in either the elastic lamella or intra-lamellar SMCs and matrix can set into motion a cascade of possible progressive damage that can manifest as intramural delamination. It appears further that this progressive damage results in a positive feedback loop, with lamellar damage, intra-lamellar SMC damage, and intra-lamellar swelling due to pooled GAGs driving each other. Even small defects can set this insidious feedback loop into motion and increased distending pressures can exacerbate the situation. Consistent with the latter, patients at risk of thoracic aortic dissections are counseled to avoid strenuous exercises that acutely raise blood pressure, and anti-hypertensive medication is commonly prescribed for these patients.

Although the SPH approach allows one to examine in detail both different loading conditions and many peculiar histomechanical characteristics that associate with aortic dissection, our model is yet limited in a number of ways. A primary limitation of the current model is the assumed quarter symmetry with period boundary conditions, which decreases computational expense considerably. Given that the defects considered – loss of elastic fiber integrity, SMC dysfunction, loss of medial collagen, and GAG accumulation – are highly localized, it does not appear that our focus on a portion of the wall compromised any of our results or conclusions. Extension of the model to a full aortic cross-section is straightforward in principle, but will increase computational expense. On average, the current sequential simulations (e.g., steps I-IV in Fig. 4 for each pool size and pressure) took 20 + hours per case when running on 128 cores of an Intel Xeon E5-2660 CPU at 2.6 GHz with MPI parallelization. Computational expense is thus an important consideration in building micro-scale models, whether via SPH or other approaches. Another limitation is that we did not model explicitly the connections between the SMCs and the elastic laminae or intra-lamellar collagen. Loss of such connections can result in dysfunctional mechanosensing, which appears to be fundamental to disease prevalence and progression^56,57^. We did not attempt to model mechanosensing or mechanoregulation of matrix, or the associated growth and remodeling responses, but such issues should be considered in the future as well.

In conclusion, our findings reveal new biophysical insights into the propagation of damage within the aortic media due to diverse defects, including elastic fiber fragmentation across the wall, dysfunction or apoptosis of SMCs, loss of intramural collagen, and accumulation of GAGs within the intra-lamellar regions. It appears that there is a strong interplay between cellular dysfunction (cell apoptosis and replacement with GAGs) and microstructural failures (elastic lamellae disruption) that manifests as a positive feedback loop, thus suggesting that independent of the initiating event, diverse defects can trigger a progressive deterioration of structural integrity. Simulations suggested further that the mechanical influence of SMC apoptosis is more localized at lower pressures, but can have far field effects at higher pressures. Consequently, growth and coalescence of local defects can lead to marked increases in wall stress throughout the wall that can lead to potentially catastrophic damage. There is, therefore, a need to correlate local effectors with clinical outcomes.

## Supplementary information

Supplementary Info
