# Supplementary material for: Modeling lamellar disruption within the aortic wall using a particle-based approach
Source: Sci Rep. 2019 Oct 25;9:15320. doi: 10.1038/s41598-019-51558-2 (PMC6814784; doi:10.1038/s41598-019-51558-2)
Supplement: Supplementary file 1 — Supplementary Info [file 41598_2019_51558_MOESM1_ESM.pdf]

Modeling lamellar disruption within the aortic wall using a particle-based approach

H. Ahmadzadeh[1], M. K. Rausch[2], J. D. Humphrey[1,*]

[1]Department of Biomedical Engineering,

Yale University, New Haven, CT, USA

[2]Department of Aerospace Engineering and Engineering Mechanics, Department of Biomedical Engineering, University of Texas at Austin, Austin, TX, USA

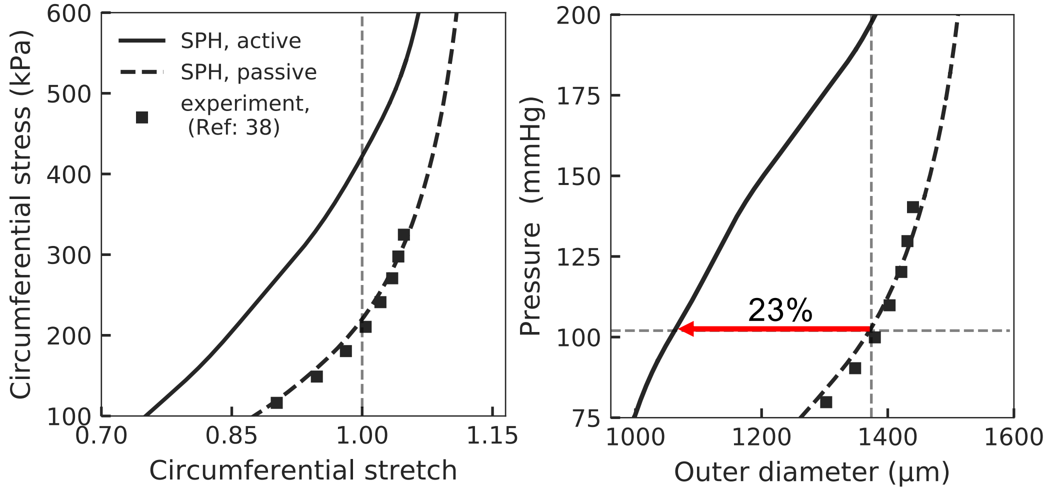

Supplemental Figure S1. Comparison of SPH simulated bulk behaviors under passive and active (SMC contraction) conditions (lines) to experimental data (symbols) for a murine descending thoracic aorta. The experimental data for the passive aorta is reported in Bersi et al., 2016 (Ref: 38). Note the good agreement within a range of physiologic pressures. At the homeostatic pressure, the SPH model predicts a 23% reduction in the outer diameter due to the SMC activation, in agreement with the experimental measurements reported in [43].

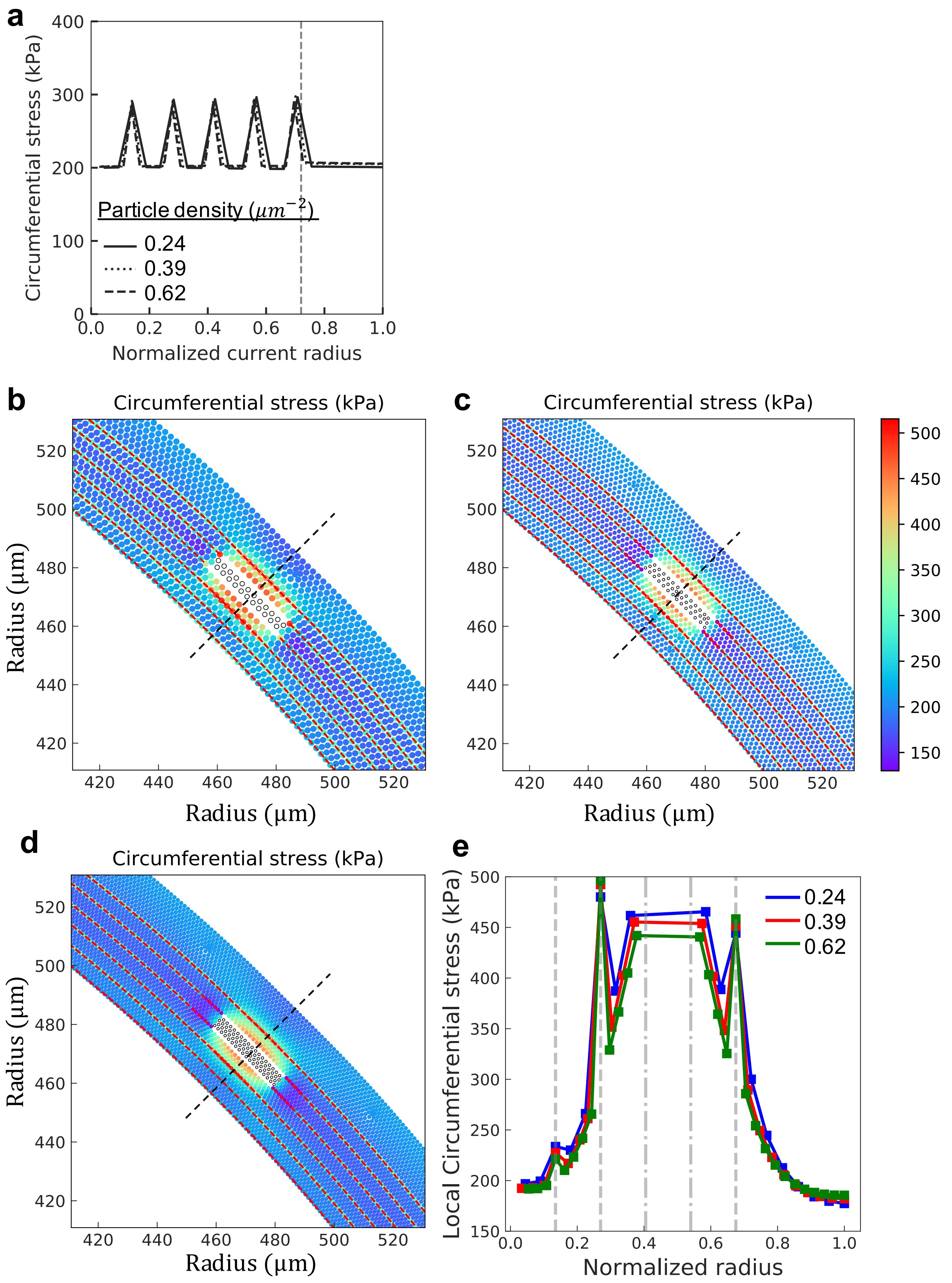
a
Circumferential stress (kPa)
Normalized current radius
Particle density (μm^{-2})
0.24
0.39
0.62
b
Circumferential stress (kPa)
Radius (μm)
c
Circumferential stress (kPa)
Radius (μm)
d
Circumferential stress (kPa)
Radius (μm)
e
Local Circumferential stress (kPa)
Normalized radius
0.24
0.39
0.62

Supplemental Fig S2. Convergence of SPH results with respect to particle resolution. Shown is the distribution of circumferential Cauchy stress in **(a)** a healthy wall and **(b,c,d)** a wall containing a pool of GAGs (open circles) with disrupted elastic lamellae (two lamellae located at the outer and inner sides of the pool) for three particle resolutions: **(b)** 0.24, **(c)** 0.39, and **(d)** 0.62 particles per μm^2. **(a)** The predicted stress in a healthy wall shows close agreement for the different particle resolutions (less than 2% difference), indicative of converged SPH results with respect to the particle density. **(e)** Spatial distributions of circumferential stress along radial cuts across the wall containing the pools of GAGs and disrupted elastic lamellae (dashed lines in **(b), (c),** and **(d)**) show similar results largely independent of particle resolution, noting that the vertical dashed lines in **(e)** locate intact elastic lamellae while the two vertical dashed-dotted lines locate the two disrupted elastic lamellae around the pools. Comparison of the stress at the elastic lamellae reveals a maximum difference of only 4-6% across the three cases. In the areas between the elastic lamellae, the three models differ mostly due to differences in the radial location of particles in the three models. As we increase particle resolution, the gradient in stress between elastic lamellae becomes more evident. The percent difference in the mean value of stress within an intra-lamellar region close to the defect was 6% between the 0.24 to 0.39 particles per μm^2 and 4% between the 0.39 to 0.62 particles per μm^2. Although we attempted to use the same size and shape of the defects in each simulation, inevitable differences arise due to the different density of particles that define the defect, which also affects the results. We thus concluded that, overall, the 0.24 particles per μm^2 was sufficient for our purposes of identifying biophysical mechanisms, not particular threshold values for damage. Note that stresses are plotted transmurally, across the defect and not at the “tip” of the defect.
